# Applications of off-gas mass spectrometry in fed-batch mammalian cell culture

**DOI:** 10.1007/s00449-019-02242-2

**Published:** 2019-11-11

**Authors:** Hai-Yuan Goh, Michael Sulu, Haneen Alosert, Graham L. Lewis, Graham D. Josland, Daniel E. Merriman

**Affiliations:** 1 The Advanced Centre for Biochemical Engineering, Department of Biochemical Engineering, University College London, Bernard Katz Building, Gordon Street, London, WC1H 0AH UK; 2 Thermo Fisher Scientific, Ion Path, Road 3, Winsford, CW7 3GA Cheshire UK

**Keywords:** Bioprocessing, Process analytics, Mass spectrometry, On-line monitoring, Process control

## Abstract

Off-gas analysis using a magnetic sector mass spectrometer was performed in mammalian cell cultures in the fed-batch mode at the 5 L bench and 50 L pilot scales. Factors affecting the MS gas traces were identified during the duration of the fed-batch cultures. Correlation between viable cell concentration (VCC) and oxygen concentration of the inlet gas into the bioreactor (O_2_-in) resulted in *R*^2^ ≈ 0.9; O_2_-in could be used as a proxy for VCC. Oxygen mass transfer (kLa) was also quantified throughout the culture period with antifoam addition at different time points which is shown to lower the kLa. Real-time specific oxygen consumption rate (*q*O_2_) of 2–20 pmol/cell/day throughout the bioreactor runs were within the range of values reported in literature for mammalian cell cultures. We also report, to our knowledge, the first instance of a distinct correlation between respiration quotient (RQ) and the metabolic state of the cell culture with regard to lactate production phase (average RQ > 1) and consumption phase (average RQ < 1).

## Introduction

Process analytical technology (PAT) is used by biopharmaceutical companies to ensure that product quality is maintained, defined by the process’ critical quality attributes, CQAs. Different techniques such as quality by design (QbD) and multivariate data analysis (MVDA) are employed, these technologies allow companies to find optimal conditions and test different parameters efficiently to reduce processing time, cost, and achieve their aimed final product.

Within the cell culture step, many techniques used to increase the information level about the cells and how they are growing, such as pH, dissolved oxygen, and temperature, are either contingent on other elements of the process or set by the user, thus not suitable for use in process analysis. Also, techniques that give more information, such as metabolite analysis are performed offline or at-line, so there is a need for more in-line analysis within cell culture.

One particular area of PAT applicable in a bioreactor that is often undervalued is the analysis of the gas phase. Given that many more measurable variables are available for analysis and have been investigated in the liquid phase, off-gas analysis appears to be less attractive. It should be noted that off-gas broadly consists of any gaseous components that go in and out of the bioreactor, and not limited to just oxygen and carbon dioxide although these two are the most commonly measured components. The use of paramagnetic and zirconium dioxide sensors for oxygen concentration determination and infra-red sensor for carbon dioxide concentration determination had been used traditionally [1–4]. These techniques have a limited detection range for the gases as well as inferior sensitivity when compared to the use of mass spectrometry (MS). While more complex in operation, mass spectrometry allows the detection and quantification of a larger variety of gaseous compounds apart from oxygen and carbon dioxide, if one chooses to do so, within a single equipment. This is in contrast to the mono-specificity of the non-MS sensors for the individual gases limited to just oxygen and carbon dioxide, the number of sensors will increase proportionally to the number of different gases of interest. Off-gas analysis using MS had been successfully demonstrated before in mammalian cell cultures at various scales [5–8]. Oxygen consumption is commonly estimated using the dynamic method or global mass balance (GMB) methods [7, 9]. The former, dynamic method, involves the increasing of the dissolved oxygen in the bioreactor to a set level before turning off the oxygen supply and allowing the dissolved oxygen to be consumed by the cells. The latter, GMB method, only requires the gas flow rates and oxygen concentration in the inlet and outlet streams.

There are several attributes of the inlet and respiratory gas streams of mammalian cell cultures that make the gas analysis challenging compared to microbial cells; the flow rate of inlet gas, especially to small reactors, is typically much lower than that of microbial fermentation, sometimes approaching the minimum requirements of the sample inlet for the mass spectrometer. This can require longer sample stream switching times, fortunately the very long batch processing times and lower respiration rates associated with mammalian cell cultures tend to not require very high frequency of analysis. In this study data were gathered from each sample stream at a frequency of once per minute which was deemed quite satisfactory.

In microbial fermentations, the feed gas composition is relatively constant—either air or air enriched with oxygen. In mammalian cell fermentations, the feed gas composition is a frequently changing mixture of several compounds (e.g., nitrogen, oxygen and carbon dioxide); concentrations of oxygen and carbon dioxide in this study varied from close to zero to 100%. This requires that the gas analysis mass spectrometer has a very wide dynamic range and is both stable and linear over this wide range.

The low respiration rate of mammalian cells compared to microbial cells yields much smaller differences in oxygen and carbon dioxide concentrations in the outlet gas compared to the inlet gas, this places a further demand on the gas analysis spectrometer that the measurement must have a very high degree of short term precision to observe these very small differences from which useful oxygen uptake rate (OUR), carbon dioxide evolution rate (CER), and respiration quotient (RQ) are derived. The use of bicarbonate as the pH buffer in culture media together with the fact that carbon dioxide is fairly soluble in aqueous media makes the determination of biotic and abiotic evolution of carbon dioxide from the system challenging [10]. A literature study summarised by Goudar et al. [7] showed that the majority of off-gas analyses have been performed in perfusion or continuous modes in bioreactors and in batch mode for culture vessels less than 1 L. It is not surprising to find that to be the case as perfusion cultures generally facilitate off-gas analysis by being in steady-state conditions and that batch cultures are more easily maintained in terms of the lack of feeding and their simplistic operations. In contrast, while being similar to batch culture in terms of varying growth and metabolic states throughout the culture, fed-batch culture has the added complication of feed additions accompanied by volume changes. A search for more recent publications resulted in two instances where off-gas analysis was employed in fed-batch bioreactor cultures with one using mass spectrometry (MS) [1] and the other with a combination of non-MS based sensors [4].

Off-gas analysis using mass spectrometry, MS, applies mass balancing principles and correlates them to the evolution and utilisation of gases focusing on carbon dioxide and oxygen (constituents of cellular respiration) throughout the duration of the cell culture. Therefore, the raw data can be compiled to infer different parameters such as respiratory quotient (RQ) which gives the ratio of carbon dioxide produced to that of oxygen consumed. The RQ value is then correlated to the metabolite and viable cell count to deduce how much of the substrates and by-products were produced and how they lead to changes in the profile of the cell culture.

In this study, we present data on off-gas analysis of mammalian cell cultures operated in fed-batch mode at bench and pilot scales. An insight into the factors affecting the MS gas traces is provided along with discussions on the significance of the various parameters derived from the off-gas analysis. We also report the first instance of a distinct correlation between RQ and the metabolic state of the cell culture with regards to lactate production or consumption.

## Materials and methods

### Experimental set-up

**Fig. 1:**
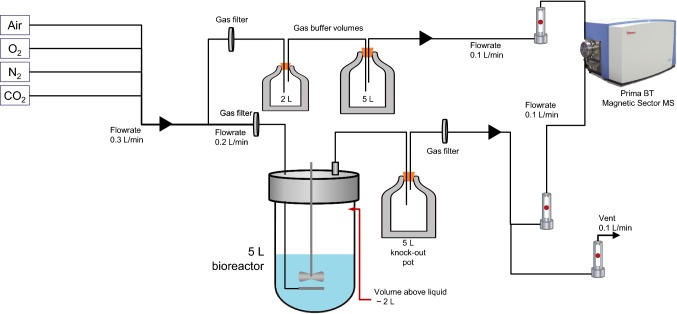
Schematic representation of the set-up of the off-gas MS analyser in the 5 L STR system

**Fig. 2:**
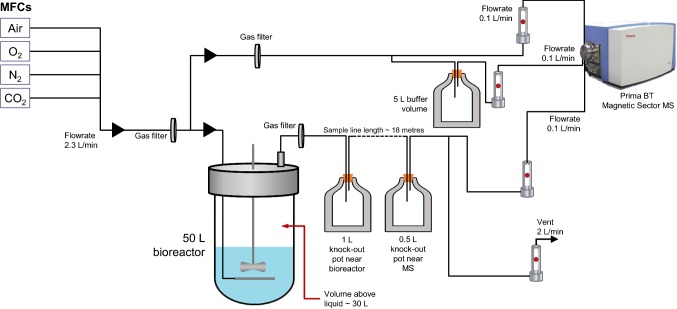
Schematic representation of the set-up of the off-gas MS analyser in the 50 L SUB system

A GS-CHO cell line that expresses the chimeric Ig4 monoclonal antibody (Mab) cB72.3 (CY01) was provided by Lonza Biologics (Slough, UK). The seed train for both the 5 L STR (Biostat BDCU, Sartorius) and 50 L SUB (BIOSTAT STR 50, Sartorius) fed-batch process involves initial expansion in shake flasks (Corning). The inoculum from the shake flasks was used for the 5 L STR experiments (two replicates). The 5 L STR was inoculated at 0.3 × 10^6^ cells/mL with an initial working volume of 2.5 L. The 50 L SUB experiment (one replicate) had its N-1 step in a 5L STR and the inoculum from the 5 L STR was used to inoculate the 50 L SUB at 0.3 × 10^6^ cells/mL with an initial working volume of 25 L. Basal- and feed media used were CD CHO (Gibco, Life Technologies) and CHO CD EfficientFeed™ B (Gibco, Life Technologies), respectively. Temperature was maintained at 37 °C ± 0.1 °C. pH was controlled at pH 7.2 ± 0.1 by sparging in carbon dioxide (CO_2_) gas and addition of base (1 M NaOH). Dissolved oxygen was maintained at 30% ± 1% air saturation using a blend of compressed air, oxygen, and nitrogen, all at 0.5 bar; mass flow controllers were present in the 50 L SUB system but not the 5 L STR system. Initial overall gas flow rate in the 5 L STR and 50 L SUB was 0.08 VVM and 0.092 VVM, respectively. Scale-up between the two systems was carried out using constant volumetric power input. Agitation and aeration in the 5 L STR were provided by a horse shoe sparger and 1 × three-bladed, pitched (45°) impeller at 230 rpm; in the 50 L SUB, a ring sparger and two three-blade segment impellers at 123 rpm were used. Addition of 1% (v/v) antifoam solution (antifoam C emulsion, Sigma Aldrich) was made at the discretion of the operator. A summary of the operating parameters was shown in Table 1. Daily monitoring was done with samples being taken and measured for viable cell concentration, viability, nutrients and metabolites, and Mab titre. Cell concentration and viability were determined by the using a Vi-cell™ XR Cell Viability Analyser (Beckman Coulter, High Wycombe, UK). Nutrients and metabolites were analysed using the BioProfile FLEX Analyzer (Nova Biomedical UK, Innovation House, Cheshire, UK). Quantification of the mAb was achieved using a 1 mL HiTrap Protein G column (GE Healthcare) on an Agilent 1200 high performance liquid chromatography (HPLC) machine (Agilent Technologies). Glucose level was used as the indicator to initiate feed. Subsequently, bolus feeding was performed once per day until the end of the culture. The bolus feed volume was 10% of initial culture volume for the first three days with subsequent days being only 5%. Gas streams going in and out of the bioreactor were connected to a magnetic sector process mass spectrometer for off-gas analysis (Thermo Scientific, Prima BT Mass Spectrometer). The configurations of the experimental set-up with respect to the use of the off-gas MS analyser are depicted in Figs. 1 and 2 for the 5 L and 50 L scale, respectively. Prior to use for cell culture the configurations were tested by assessing forced perturbations, to assess the correct buffer volumes and path lengths and minimise data lag from the reactors.

**Table 1:** Summary of operating parameters of 5 L STR and 50 L SUB

Conditions/parameters	2 × 5L stirred-tank reactor (STR)	50L single-use bioreactor (SUB)
Initial working volume (L)	2.5	25
Inoculation density (× 10^6^ cell/mL)	0.3	
Temperature (ºC)	37	
pH control	7.2 ± 0.1 via carbon dioxide (CO_2_) gas and addition of base (1 M NaOH)	
Dissolved oxygen (% air saturation)	30	
Sparger/impeller	horse shoe sparger; 1 × three-bladed, pitched (45º) impeller	Ring sparger, 2 × three-bladed, pitched impeller
Antifoam (% v/v)	Antifoam C, 1%

### Off-gas analysis

Further processing of the data from the off-gas MS analyser could be performed to elucidate more details about cell cultures in bioreactors. The following parameters were derived from the raw off-gas data: oxygen uptake rate (OUR), kLa (for O_2_), specific oxygen consumption rate (*q*O_2_), carbon dioxide evolution rate (CER), specific carbon dioxide production rate (*q*CO_2_) and respiration quotient (RQ).

1 $${\text{OUR}} = \left( {\frac{{F_{{\text{bioreactor,in}}} }}{{F_{{\text{MS,in}}} }}} \right)\left( {F_{{\text{MS,in}}} } \right)\left( {\frac{{{\text{OxC}}}}{100}} \right)\left( {\frac{1}{{V_{{{\text{bioreactor}}}} }}} \right),$$

2 $$k_{{\text{L}}} a = \frac{{{\text{OUR}}}}{{[{\text{O}}_{2}^{*} \left] { - [{\text{O}}_{2} } \right]}},$$

3 $$q{\text{O}}_{2} = \frac{{{\text{OUR}}}}{{{\text{VCC}}}},$$

4 $${\text{CER}} = \left( {\frac{{F_{{\text{bioreactor,in}}} }}{{F_{{\text{MS,in}}} }}} \right)\left( {F_{{\text{MS,in}}} } \right)\left( {\frac{{{\text{CDC}}}}{100}} \right)\left( {\frac{1}{{V_{{{\text{bioreactor}}}} }}} \right),$$

5 $$q{\text{CO}}_{2} = \frac{{{\text{CER}}}}{{{\text{VCC}}}},$$

6 $${\text{RQ}} = \frac{{{\text{CER}}}}{{{\text{OUR}}}},$$

where $$F_{{\text{bioreactor,in}}}$$,$$F_{{\text{bioreactor out}}}$$$$F_{{\text{MS,in}}}$$, $$V_{{{\text{bioreactor,}}}}$$$$[{\text{O}}_{2}^{*} ]$$, $$[{\text{O}}_{2} ]$$ and VCC are the gas flow rate into the bioreactor in mL min^−1^, the gas flow rate out of the bioreactor in mL min^−1^, gas flow rate into the MS analyser in mL min^−1^, volume of culture in bioreactor in L, oxygen concentration in the inlet gas stream in µmol L^−1^, oxygen concentration measured in the culture in µmol L^−1^, viable cell concentration at the particular instance in cellmL^−1^, respectively. $${\text{OxC}}$$ and $${\text{CDC}}$$ are derived parameters obtained from the GasWorks software associated with the MS, that are derived using Eqs. 7–14 below and applicability was reported by Van [11]

7 $${\text{OUR}} = \left( {\left( {\% {\text{mol}}\; {\text{of}} {\text{O}}_{{{\text{2in}}}} \times F_{{\text{bioreactor in}}} } \right) - \left( {\% {\text{mol}} O_{{\text{2 out}}} \times F_{{\text{bioreactor out}}} } \right)} \right)/100,$$

8 $${\text{CER}} = \left( {\left( {\% {\text{mol}}\;{\text{ of}}\; {\text{CO}}_{{\text{2 out}}} \times F_{{\text{bioreactor out}}} } \right) - \left( {\% {\text{mol CO}}_{{\text{2 in}}} \times F_{{\text{bioreactor in}}} } \right)} \right)/100.$$

As measurement of flow rates can be inaccurate and the MS has the capacity to measure N_2_ accurately and as it is not consumed via respiration, then:

9 $${\text{\%mol}}\;{\text{ of}}\; {\text{N}}_{{\text{2 in}}} \times F_{{\text{bioreactor in}}} = \% {\text{mol }}\;{\text{of}} \;N_{{\text{2 out}}} \times F_{{\text{bioreactor out}}} ,$$

10 $$F_{{\text{bioreactor out}}} = \left( {\frac{{{\text{\%mol }}\;{\text{of}}\; {\text{N}}_{{\text{2 in}}} \times F_{{\text{bioreactor in}}} }}{{{\text{\%mol}} \;{\text{of}}\; {\text{N}}_{{\text{2 out}}} }}} \right) .$$

Substituting this into the classic CER and OUR formulae and then simplifying, we get:

11 $${\text{CER}} = \left( {{\text{\%mol}}\; {\text{of}} \;{\text{CO}}_{{\text{2 out}}} \times \left( {\frac{{{\text{\%mol}} \;{\text{of}}\; {\text{N}}_{{\text{2 in}}} }}{{{\text{\%mol }}\;{\text{of}}\; {\text{N}}_{{\text{2 out}}} }}} \right)} \right) - \left( {{\text{\%mol}} {\text{CO}}_{{\text{2 in}}} } \right) \times F_{{\text{bioreactor in}}} /100,$$

12 $${\text{OUR}} = \left( {\% {\text{mol}} \;{\text{of}} \;{\text{O}}_{{\text{2 in}}} - \left( {\% {\text{mol}} \;{\text{of}} \;{\text{O}}_{{\text{2 out}}} \times \left( {\frac{{\% {\text{mol}}\;{\text{ of}}\; {\text{N}}_{{\text{2 in}}} }}{{\% {\text{mol}}\;{\text{ of}}\; {\text{N}}_{{\text{2 out}}} }}} \right)} \right.} \right) \cdot \left( {{\raise0.7ex\hbox{${F_{{\text{bioreactor in}}} }$} \!\mathord{\left/ {\vphantom {{F_{{\text{bioreactor in}}} } {100}}}\right.\kern-\nulldelimiterspace} \!\lower0.7ex\hbox{${100}$}}} \right) .$$

Therefore substituting and simplifying again:

13 $${\text{RQ}} = \left( {\frac{{{\text{\%mol of CO}}_{{\text{2 out}}} \times \left( {\frac{{{\text{\%mol }}\;{\text{of }}\;{\text{N}}_{{\text{2 in}}} }}{{{\text{\%mol }}\;{\text{of }}\;{\text{N}}_{{\text{2 out}}} }}} \right) - {\text{\%mol CO}}_{{\text{2 in}}} }}{{\left( {{\text{\%mol O}}_{{\text{2 in}}} - \left( {{\text{\%mol O}}_{{\text{2 out}}} \times \left( {\frac{{{\text{\%mol }}\;{\text{of }}\;{\text{N}}_{{\text{2 in}}} }}{{{\text{\%mol }}\;{\text{N}}_{{\text{2 out}}} }}} \right)} \right)} \right)}}} \right),$$

where the gasworks software labels the numerator CDC and the denominator OxC, and

14 $${\text{RQ } = \text{ }}\left( {\frac{{{\text{CER}}}}{{{\text{OUR}}}}} \right){ = }\left( {\frac{{{\text{CDC}}}}{{{\text{OxC}}}}} \right).$$

## Results and discussions

### Factors affecting MS gas traces

**Fig. 3:**
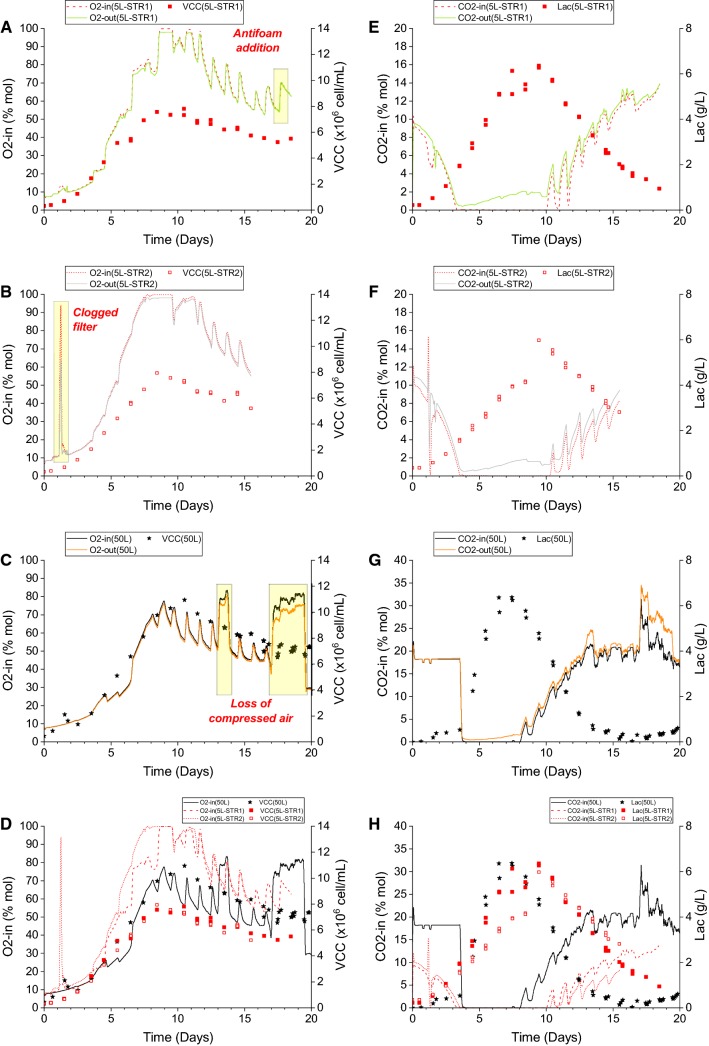
Overlay of real-time off-gas traces from the MS and offline cell culture data. **a** O_2_-in and O_2_-out with VCC for 5 L STR1, **b** O_2_-in and O_2_-out with VCC for 5 L STR2, **c** O_2_-in and O_2_-out with VCC for 50 L SUB, **d** Overlay of **a**–**c**, **e** CO_2_-in and CO_2_-out with lactate concentration for 5 L STR1, **f** CO_2_-in and CO_2_-out with lactate concentration for 5 L STR1, **g** CO_2_-in and CO_2_-out with lactate concentration for 50 L SUB and **h** Overlay of **e**–**g**

It should be noted that while no control loop with respect to the MS gas traces was applied in these experiments, observations were made on the MS gas traces during the fed-batch bioreactor runs. This served to show the difference between data noise from a typical fed-batch culture in the bioreactor and distinct observations made by the MS. Several actions and events during the cell culture that had the potential to affect the off-gas MS traces had been identified. They are summarised in Table 2 and the actual measurable outcomes of these actions on the gas traces are shown in Fig. 3.

**Table 2:** Summary of observations on the off-gas MS gas traces by the various events during cell culture

Actions /Events	Observations on MS traces		References
Routine sampling	No observable effect		Observed in all sampling actions
Antifoam addition	Immediate effect	Disturbance to gas traces observed	Figure 3a
Feed media (<10 °C) bolus addition	Immediate effect	Disturbance to gas traces observed	Observed in all feeding actions
Clogged sterile exhaust/venting filter	Immediate effect	Disturbance to gas traces observed	Figure 3b
Loss of compressed air	Immediate effect	Disturbance to gas traces observed	Figure 3c

Routine sampling (for offline analysis) from both the 5 L and 50 L systems did not have any observable effect on the gas traces. The sampling volume for the 5 L and 50 L systems was 20 mL and 30 mL, respectively. Compared to the bulk volume of the culture media, the removal of the sample volumes was inconsequential to the gas traces as detected by the off-gas analyser. Both the separate addition of antifoam and feed media were shown to cause an immediate spike in the O_2_ gas traces with a proportional decrease in the CO_2_ and N_2_ gas traces (N_2_ data not shown). This phenomenon supported the hypothesis that the use of antifoam would reduce the oxygen mass transfer coefficient (kLa) in the culture and as such, an inlet gas supply with higher proportion of O_2_ was needed. This was evident in the spike observed in the O_2_ gas traces. These O_2_ spikes were thought to have been masked during the exponential phase of the cultures (before ~ Day 10) because of the rapid proliferation of cells and their subsequent increase in O_2_ demand. These spikes in O_2_ were much more pronounced in the stationary phase of the cultures (after ~ Day 10) and onwards where cell density was decreasing. The reduced demand for O_2_ enabled the O_2_ spikes from antifoam addition to be more obvious. A similar O_2_ spike phenomenon was also observed when feed media was added. While the exact components of the feed media were not known publicly as it was a proprietary media, it was believed that EfficientFeed™ B contained surfactants, like Pluronic, and possibly other compounds that had a direct impact on the kLa. This could possibly account for the spike in O_2_ during feed addition as observed during antifoam addition.

Interestingly, two significant events were also captured during the cell cultures. First, in the 5 L scale bioreactor run, a clogged venting filter was detected by the MS gas traces. This detection came in the form of a significantly large spike in O_2_ (with corresponding dips in other gas components). This particular event was picked up by the MS software overnight when no operator was present. Upon checking of the MS gas traces in the morning, a troubleshooting effort was conducted and the clogged venting filter was identified as the root cause of the massive gas spike. Subsequent replacement of the filter under aseptic conditions allowed the culture to proceed as normal. The culture process had been successfully recovered from a fault that had been detected early by the MS. To the knowledge of the authors, such a fault would not have been discovered without the real-time off-gas data from the MS. Second, during the 50 L run, there were instances when the compressed air supply to the bioreactor was lost and the off-gas MS analyser picked up the changes in the gas composition. With the compressed air supply diminished, the 50 L MFC system was able to switch to a different blend of gas to supply the oxygen for the cultures. This newer blend would be one with pure oxygen instead of the usual compressed air supplemented with oxygen. There was no change in the DO reading from the Multiple Fermenters Control System (MFCS) control panel of the 50 L system which had been set at 30% DO with respect to air saturation. This meant that, on both occasions where the compressed air supply was interrupted, the culture was not compromised in any meaningful way as sufficient oxygenation was still available. This situation suggested that the use of an off-gas MS analyser would be a suitable backup for the MFCS system given that the former was an orthogonal way of quantifying the proportion of gas to be delivered into the bioreactor. The authors would acknowledge that a complete replacement of the MFCS system with the off-gas MS analyser would be impractical due to the other functions of the MFCS system but that the analyser would be an ideal backup specifically for the gassing strategy. In addition if the MS was to be used for control, the observations shown in Table 2 should be considered important to be acknowledged in a control strategy.

**Fig. 4:**
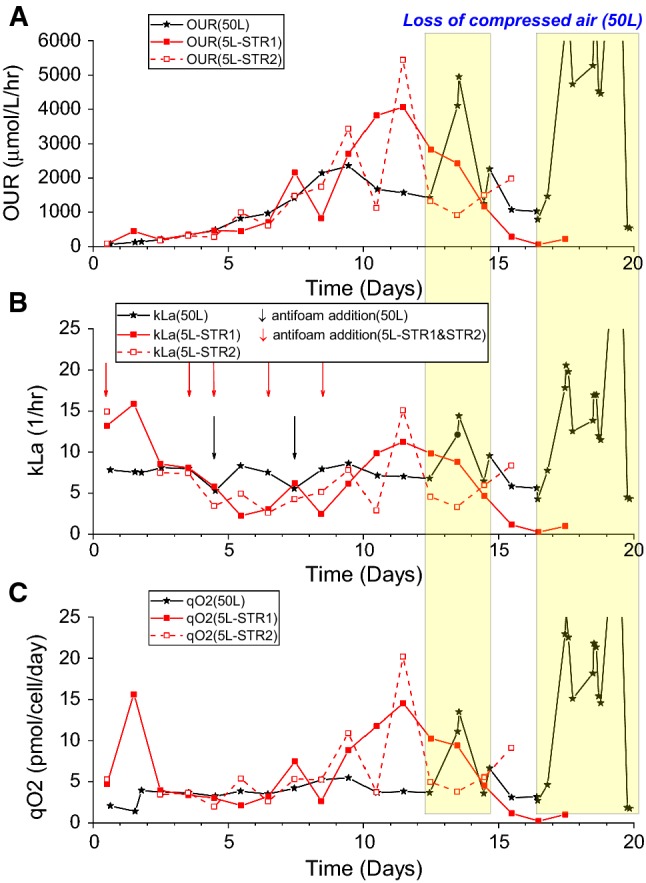
Oxygen-related parameters derived from the off-gas data of the duplicate 5 L STR runs and a single 50 L SUB run. **a** OUR values, **b** kLa values were determined after the antifoam additions and **c***q*O_2_ values; loss of compressed air was experienced during Day 12–15 and Day 16–20 only in the 50 L SUB culture

In general, the off-gas data from the 50 L run were less noisy as shown by the smoother trend lines while the 5 L runs were particularly noisy at the beginning of the cultures. Given that the growth (VCC) profiles during the beginning were similar in both scales (Fig. 3d), it was thought that the larger fluctuations in the off-gas data in the 5 L were not related to the cultures themselves but that the gas flow rates and compositions going into the MS were the more plausible causes. In the 5 L bioreactor set-up, gas flow rates were particularly difficult to maintain at the fixed flow rate as per the bioreactor feeding and operating strategies. This was due to the hardware design of the control system where individual gas sources (compressed air, O_2_, CO_2_ and N_2_) were taken directly from the respective gas taps with no individual mass flow controllers or any means to modulate the composition of the final gas mixture into the bioreactor based on the available gas sources. It should be noted that there was a single analogue flow meter to control the bulk flow rate right before the gas was delivered into the bioreactor. While there were control loops in place for process set points like pH and DO, the gases used to effect those changes were delivered in a pulsating manner (controlled via solenoid valve) where only a single gas source was engaged at any given time. For instance, when the measured DO was not at the set point, only 1 of the 3 gases for DO control, compressed air, O_2_ and N_2_, would be engaged while the valves for all other gases would be closed off. This meant that there was no pre-mixing of gases outside of the bioreactor. This one-at-a-time (on/off) gas delivery system would cause fluctuations in the analogue flow meter which prevented the volumetric gas flow rate into the 5 L bioreactors to be kept constant. This would further complicate the issue of ensuring a constant gas flow rate was being diverted from the gas inlet of the bioreactor to the MS for off-gas analysis. This was in contrast to the 50 L system which was, in fact, a newer platform compared to the 5 L system. The 50 L system had individual mass flow controllers for each of the 4 gas sources used in the 5 L, and the flow of each gas was automatically blended and controlled by the system. This meant that at any given time, multiple gas sources could be engaged simultaneously to deliver gas mixture of a given composition at the stipulated final gas flow rate into the 50 L bioreactor. This also meant that a constant gas flow rate could always be diverted away from the 50 L SUB and into the MS for off-gas analysis. This smooth transition during process control changes like DO and pH brought about using the different gas sources was translated to the less noisy data for the 50 L system than the 5 L system when off-gas data was processed as shown in Figs. 4 and 6.

### Derived parameters from off-gas data

OUR, kLa and *q*O_2_ were determined for the entire culture durations for the 5 L and 50 L runs. For the 50 L data, it should also be pointed out that the lack of compressed air occurred during Day 12–15 and Day 16–20 as shown by the highlighted blocks in Fig. 4; the two 5 L runs did not experience any loss of compressed air. The combined data from 5 and 50 L bioreactor runs presented in Fig. 4 revealed that there were discernible trends across the different scales and the different parameters.

**Fig. 5:**
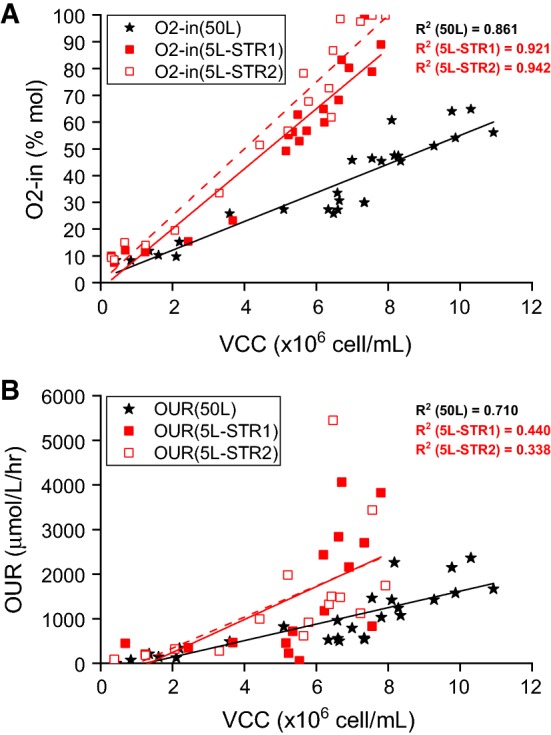
Correlation of VCC and off-gas data from the duplicate 5 L STR runs and single 50 L SUB run. **a** VCC vs O_2_-in. **b** VCC vs OUR

Across both the 5 L and 50 L scales, O_2_-in data as shown in Fig. 4a indicated that O_2_-in correlated with the VCC profile of the cultures as shown in Fig. 3d. This was not surprising as O_2_-in should increase with oxygen consumption (or OUR), which in turn would increase when more cells were present and vice versa. The correlations between the VCC and O_2_-in for the 5 L and 50 L runs were all ~ 0.9 (Fig. 5a). Unlike the OUR, O_2_-in was a raw data measurement that was independent of the other measurements like bioreactor volume or gas flow rate The correlation between OUR and VCC, however, was non-existent for the 5 L system and weak for the 50 L system (Fig. 5b). This was thought to be due to the noisier OUR data generated from the 5 L system and the fairly inconsistent gas flow rates present inherent to that system as described earlier; OUR calculation required the use of gas flow rates. While the 50 L run had an *r*^2^ value of 0.710 compared to the 5 L runs at 0.440 and 0.338 based on the VCC-OUR data, the former’s correlation was still relatively weak. This was likely due to the inaccuracies attributed to the determination of bioreactor volume and the lack of data points from the VCC measurements. This also suggested that O_2_-in would be a more reliable online indicator of VCC changes in a cell culture and potentially a better parameter for process control.

The kLa could be easily determined from the known gas flow rates and oxygen concentration in the inlet and outlet gas streams of the bioreactors. This was in contrast to the traditional dynamic gassing-in/out method where perturbations to oxygen supply were necessary to determine the kLa at a particular instance during the culture; performing such perturbations regularly during a bioreactor run could be detrimental to the cells. The kLa data suggested that the initial kLa in the 5 L bioreactors (~14 h^−1^) were higher than the 50 L bioreactor (~7.5 h^−1^). The reduction of kLa as a result of the antifoam addition was expected and the off-gas analysis was able to capture that. The drop in kLa from antifoam addition was also observed in the 5 L runs though it was not as comparable between the two replicates (Fig. 4b). In addition, analysis of the change in volume with the change in kLa showed no significant difference, even at the larger scale where there is an extra impeller utilised.

The *q*O_2_ trends were comparable across the different bioreactor runs (Fig. 4c). A relatively constant *q*O_2_ was maintained for the first half of the cultures while a relatively higher *q*O_2_ was observed in the second half of the cultures. One hypothesis to account for this change would be the metabolism shift from lactate production to consumption. During periods of rapid accumulation of lactate, glycolysis was the pre-dominant pathway for the cells where relatively lower amount of oxygen was required. The shift to lactate consumption was likely an indication that oxidative phosphorylation, which actively required oxygen as the final electron donor in a series of redox reactions, became the main mechanism of generating energy. Hence, the *q*O_2_ became relatively higher just when the lactate shifted to a net consumption behaviour. This was observed in the 50 L run and 5 L runs after Day 7 and Day 9, respectively, (Fig. 4c) where the days also coincide with the shift in lactate metabolism in the respective runs (Fig. 3h). It was interesting to note that from Day 10 onwards, the *q*O_2_ was ~ 3 time as high in the 5 L runs than the 50 L run even though the cell density was on average higher in the latter. This phenomenon could not be accounted for at the moment, but it suggested that perhaps there was a different physiological environment in the 5 L system than the 50 L system that caused a higher *q*O_2_ in the former systems. The range of *q*O_2_ values is greater than has been previously reported, however these tend to have been reported with perfusion systems, and that may have an impact, as well as the method for measurement used in previous studies.

**Fig. 6:**
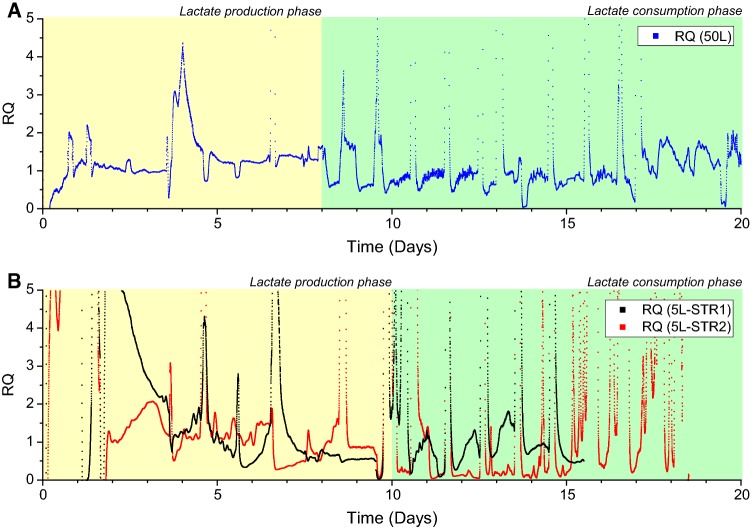
Respiration quotient profiles during the duplicate 5 L STR runs and single 50 L SUB run as determined from the off-gas data with the lactate production phase and lactate consumption phase highlighted in yellow and green, respectively. **a** RQ data from 50 L and **b** RQ data from 5 L. The detailed lactate profile of each run can be found in Fig. 3e–g

It should also be noted that in the period with no CO_2_ gassing into the bioreactors, CO_2_ could be detected in the exhaust gas from the cell cultures (Fig. 3e–g). The highest proportion of CO_2_ in the gas streams that exited the bioreactors was ~ 2% mol. This production of CO_2_ from the system was likely due to the biotic contribution of CO_2_ evolution from cellular respiration. Abiotic contribution of CO_2_ from the bicarbonate buffer equilibrium could favour CO_2_ production. However, the fact that NaOH, a strong base, was added during the period when lactate concentration was high and no CO_2_ was gassed into the bioreactor suggested that the bicarbonate equilibrium would shift towards formation of bicarbonate (HCO_3_^−^) ions than the carbonic acid (H_2_CO_3_). This would result in the biotic contribution to be the only source of CO_2_ evolution during this particular period. CER and *q*CO_2_ were only determined during this particular period of no external CO_2_ gassing into the bioreactors. While the data was not shown, the *q*CO_2_ appeared to be comparable among the 3 different runs across the two different scales at ~ 5 pmol/cell/day. Both the *q*CO_2_ values (~5 pmol/cell/day) and *q*O_2_ values (2–20 pmol/cell/day) as described earlier were within the range of values reported by Goudar et al. [7] who had presented the *q*O_2_ and *q*CO_2_ values from their own experimental work and collated these values from other literature. In contrast to this however, abiotic production of CO_2_ while considered less likely in this instance, would explain the noisy RQ profile as seen in Fig. 6, but this could equally be attributed to the poor OUR correlation shown in Fig. 4.

### Relationship between RQ and metabolic state of cell culture

RQ could be a convenient parameter to look at as an indicator of the metabolic state of the cell culture. The off-gas data from the MS was able to provide real-time RQ measurements (Fig. 6). It was evident that the gas spikes as a result of feed and antifoam addition, as shown previously in Fig. 4b, had also affected the RQ. It should also be mentioned that the RQ determination was based on the CER and OUR values from the MS and that the CER was corrected for neither the abiotic CO_2_ contribution from the bicarbonate in the media given that its concentration was not known nor the relatively higher solubility of CO_2_ than O_2_. Despite so, general trends could still be gleaned from the data while keeping in mind that there would be slight under/over estimation of the CER term and consequently the RQ term.

The RQ profiles for the duplicate 5 L runs (Fig. 6b) were not a true replicate of one another. This was likely an indication that there were actual physical differences in the way the gases were delivered into each of the 5 L bioreactors. The main suspect would again be the gas flow rates, both into the bioreactors and into the MS, which had been observed to fluctuate with the changing gas demands from each of the cultures due to the use of the analogue flow meter and the on/off nature of the gas delivery system found in the 5 L system. In addition, both reactors are fed by the same gas source in series, so the effect of the change in inlet gas flow rate can change depending on the gases that are being requested by each individual reactor. These data noise contributors were less of an issue in the 50 L system that allowed clearer interpretation of its RQ profile.

The more interesting and perhaps more relevant observation on the RQ profiles was the correlation between the state of the lactate metabolism (production or consumption) and the approximate averaged-out RQ values during each metabolic state. The trend was more noticeable in the 50 L run than the duplicate 5 L runs. If the large spikes/dips of the RQ profiles contributed by the feed and antifoam additions as discussed before were ignored, the RQ value, on average, was > 1 during the lactate production phase and was < 1 during the lactate consumption phase (Fig. 6a). Pure utilisation of a carbon source like glucose would result in a RQ value of 1 while oxidation of the more reduced molecules like amino acids and fatty acids would result in RQ < 1 as more oxygen would be needed to completely oxidise these molecules. During the lactate production phase, glycolysis would be the main metabolic pathway as indicated by the high specific glucose consumption rate (data not shown) and relatively lower *q*O_2_ and consequently a higher RQ. On the other hand, during lactate consumption phase, oxidative phosphorylation involving a higher TCA activity was represented by a lower specific glucose consumption rate (data not shown) and relatively higher *q*O_2_ which ended up with a lower RQ. This relationship between a high- and low glycolytic flux with lactate production and consumption, respectively, had been previously demonstrated in shake flasks and deep well plate experiments [12]. Multivariate analysis of more than 200 manufacturing production bioreactor runs with varying lactate production/consumption behaviours had also suggested that a shift from lactate production to consumption had more to do with a lowered glycolytic flux than any other parameters [13].

While also taking into consideration that there were inherent variations in the RQ values due to the inconsistent gas flow in the 5 L runs, this implied that relatively distinct metabolic states could be determined based on the RQ profiles. Even though the CER term could not be effectively corrected for, the RQ data may correlate well with the metabolic states, especially in the 50 L where the off-gas signals were less noisy. To the knowledge of the authors, there had been no experimental data published pertaining to RQ and different metabolic states. RQ values were rarely determined in mammalian cell cultures and even much lesser in non-perfusion modes of cultivation due to the lower peak VCC and non-steady state nature found in those modes. It was also believed that the unique RQ-lactate metabolism relationship was successfully captured in part due to the lactate profile of the cell cultures performed. The cultures had relatively long and distinct periods of lactate production followed by lactate consumption in both the 5 L and 50 L bioreactors. This facilitated the detection of the change in RQ during the respective periods of lactate production and consumption. To fully assess whether RQ would change as a function of metabolism in such a defined way (as would theoretically be expected) more experimentation would be needed.

As the system currently stands, the ability to deconvolute CO_2_ from metabolism or other sources mean that both CER & RQ would always have an element of uncertainty. With this in mind, the authors believe that any O_2_-related parameters would be a better parameter to use with respect to process analysis and control. Furthermore, due to the calculation of OUR requiring the accurate knowledge of other parameters, the simple parameter of O_2_-in, which appeared as a proxy for VCC (Fig. 5), could be used to both measure and control the state of the cell cultures, especially those with high cell density. Recently, it had also been demonstrated that the correlation between the cumulative oxygen transfer rate and the cumulative glucose consumed could be used as an advanced control strategy to maintain glucose level in the media at a given set point [14].

### Potential applications of off-gas analysis

There are several applications of off-gas MS analyser that have not been demonstrated in the context of the experiments presented herein. They are thought to be feasible as future experimentations or even implementations in real world manufacturing settings. Such potential applications could include:

1. Magnetic sector MS can detect non-typical gas components like hydrogen sulphide (H_2_S) down to the ppm range and be used as indicators of cellular metabolism stress or product quality attributes.
2. Early detection of microbial contamination based on off-gas traces alone could minimise wait time for critical decision making for batch termination.
3. Evaluation of batch to batch variation, within predefined specifications, for robust technology transfer across different sites and bioreactor platforms.

## Conclusions

In summary, the application of off-gas analysis using a magnetic sector mass spectrometer had been successfully implemented in the 5 L and 50 L scales for fed-batch mammalian cell cultures. Several factors had been identified to affect gas traces from the MS although they would likely change from system to system. The real-time O_2_-in gas traces in % mol parameter correlated well with the different phases of the cell cultures and especially with the VCC. An inverse relationship was observed between the real-time CO_2_ gas traces and the lactate profile of the cultures. kLa could be derived throughout the entire culture duration given the necessary known parameters simply from the off-gas data. Furthermore, specific actions that were known to influence kLa like antifoam additions could be determined from the off-gas analysis. Finally, the observed RQ profiles appeared distinct enough to show a correlation with either the lactate production or consumption phase during the cultures. It is assumed that the use of an industrial GS-CHO cell line, would have no impact versus the use of a non-GS-CHO line, due to the different metabolisms and the technique would still be applicable.

## References

[1] Aehle M, Kuprijanov A, Schaepe S, Simutis R, Lubbert A (2011). Simplified off-gas analyses in animal cell cultures for process monitoring and control purposes. Biotech Lett.

[2] Frahm B, Blank H-C, Cornand P, Oelßner W, Guth U, Lane P (2002). Determination of dissolved CO2 concentration and CO_2_ production rate of mammalian cell suspension culture based on off-gas measurement. J Biotechnol.

[3] Lovrecz G, Gray P (1994). Use of on-line gas analysis to monitor recombinant mammalian cell cultures. Cytotechnology.

[4] Winckler S, Krueger R, Schnitzler T, Zang W, Fischer R, Biselli M (2014). A sensitive monitoring system for mammalian cell cultivation processes: a PAT approach. Bioprocess Biosyst Eng.

[5] Behrendt U, Koch S, Gooch DD, Steegmans U, Comer MJ (1994). Mass spectrometry: a tool for on-line monitoring of animal cell cultures. Cytotechnology.

[6] Eyer K, Oeggerli A, Heinzle E (1995) On-line gas analysis in animal cell cultivation: II. Methods for oxygen uptake rate estimation and its application to controlled feeding of glutamine. Biotechnol Bioeng 45(1):54–62. 10.1002/bit.260450108 18623051

[7] Goudar CT, Piret JM, Konstantinov KB (2011). Estimating cell specific oxygen uptake and carbon dioxide production rates for mammalian cells in perfusion culture. Biotechnol Prog.

[8] Oezemre A, Heinzle E (2001). Measurement of oxygen uptake and carbon dioxide production rates of mammalian cells using membrane mass spectrometry. Cytotechnology.

[9] Ruffieux P-A, von Stockar U, Marison IW (1998). Measurement of volumetric (OUR) and determination of specific (*q*O_2_) oxygen uptake rates in animal cell cultures. J Biotechnol.

[10] Bonarius HPJ, de Gooijer C, Johannes T, Georg S (1995). Biotechnol Bioeng.

[11] Van der Aar PC, Southamer AH, Van Verseveldeveld HW (1989). Possible misconceptions about O_2_ consumption and CO_2_ production measurements in stirred microbial cultures. Journal of Microbial Methods.

[12] Templeton N, Dean J, Reddy P, Young JD (2013). Peak antibody production is associated with increased oxidative metabolism in an industrially relevant fed-batch CHO cell culture. Biotechnol Bioeng.

[13] Le H, Kabbur S, Pollastrini L, Sun Z, Mills K, Johnson K (2012). Multivariate analysis of cell culture bioprocess data—lactate consumption as process indicator. J Biotechnol.

[14] Goldrick S, Lee K, Spencer C, Holmes W, Kuiper M, Turner R, Farid SS (2018). On-line control of glucose concentration in high-yielding mammalian cell cultures enabled through oxygen transfer rate measurements. Biotechnol J.

